# Case Report: Intra-procedural fatal cerebral lipiodol embolism without angiographically visible shunting during right inferior phrenic artery TACE

**DOI:** 10.3389/fonc.2026.1896410

**Published:** 2026-09-03

**Authors:** Yannan Xie, Chunyu Zhang, Chaofeng Tang, Bendong Chen

**Affiliations:** 1 Department of Hepatobiliary Surgery, General Hospital of Ningxia Medical University, Yinchuan, China; 2 Ningxia Medical University, Yinchuan, China

**Keywords:** acute ischemic stroke, cerebral lipiodol embolism, hepatocellular carcinoma, iodized oil, right inferior phrenic artery transarterial chemoembolization, transarterial chemoembolization

## Abstract

**Background:**

CLE can occur during right inferior phrenic artery TACE despite negative angiographic findings. Negative angiography should not be interpreted as excluding occult embolic pathways, and radiologic clearance of intracranial Lipiodol should not be equated with neurological recovery.

**Case presentation:**

A 51-year-old man with clinically diagnosed malignant liver tumor who developed fatal CLE during the fourth liver-directed procedure. During selective right inferior phrenic artery transarterial chemoembolization (TACE), angiography showed hepatic-dome tumor staining. While the iodized-oil emulsion was being injected, the awake patient abruptly developed bilateral limb weakness that progressed to complete loss of limb strength and impaired consciousness. Cranial CT performed immediately after symptom onset showed multiple hyperdense intracranial lesions that largely disappeared on virtual non-contrast images, supporting iodized-oil deposition. MRI-DWI demonstrated multifocal acute ischemic lesions in bilateral supratentorial and infratentorial territories, while MRA showed no major intracranial arterial occlusion. Follow-up CT on postoperative days 1 and 3 showed progressive clearance of intracranial Lipiodol deposits, but the ischemic lesions and severe neurological deficits persisted, and death was confirmed. This temporal-imaging-outcome sequence shows that radiologic Lipiodol clearance does not necessarily indicate neurological reversibility once widespread acute ischemic injury has occurred.

**Conclusion:**

CLE can occur during right inferior phrenic artery TACE despite negative angiographic findings. Negative angiography should not be interpreted as excluding occult embolic pathways, and radiologic clearance of intracranial Lipiodol should not be equated with neurological recovery.

## Introduction

Transarterial chemoembolization (TACE) is an established locoregional therapy for patients with hepatocellular carcinoma (HCC) who are not candidates for curative resection or ablation, particularly in intermediate-stage disease and selected multidisciplinary treatment settings (1–3). Iodized oil is widely used as a carrier and embolic component in conventional TACE (4). Most TACE-related adverse events include post-embolization syndrome, transient hepatic injury, biliary complications, abscess, gastrointestinal injury, pulmonary embolism, and access-site events (5, 6). In contrast, cerebral Lipiodol embolism (CLE) is extremely uncommon but may cause rapid and irreversible neurological injury (7–13).

Recent case literature has emphasized vascular lakes, visible shunting, delayed presentation, or recovery after CLE (9, 13). The present case is intentionally framed differently. It provides a complete intra-procedural and follow-up evidence chain: symptoms began during iodized-oil injection in an awake patient, embolization was stopped immediately, emergency CT/virtual non-contrast imaging demonstrated iodine-compatible intracranial deposits, RI-DWI confirmed multifocal acute ischemia showed no major arterial occlusion; and serial CT showed Lipiodol clearance without neurological recovery. This sequence highlights two practical blind spots: negative angiography may not exclude clinically relevant occult embolic pathways, and disappearance of intracranial Lipiodol density does not necessarily indicate reversibility of ischemic brain injury. This report was prepared in accordance with the CARE guideline (14).

## Case description

A 51-year-old man was admitted on August 19, 2025, because of right upper abdominal pain for five days. HCC was diagnosed clinically on the basis of chronic hepatitis B-related cirrhosis, markedly elevated alpha-fetoprotein (AFP), and characteristic findings on contrast-enhanced abdominal CT and MRI, including arterial-phase hyperenhancement and washout in the portal venous or delayed phase (3, 15). Histological confirmation was not obtained. Initial assessment showed Child-Pugh class A liver function, China Liver Cancer (CNLC) stage IIb disease, and Barcelona Clinic Liver Cancer (BCLC) stage B disease (2, 15).Multidisciplinary discussion concluded that the multifocal tumor was not suitable for curative surgery, and transarterial therapy combined with immunotherapy, targeted therapy, and reassessment for conversion therapy was recommended. Treatment risks, including ectopic embolization, were discussed with the patient and family before intervention.

**Figure 1:**
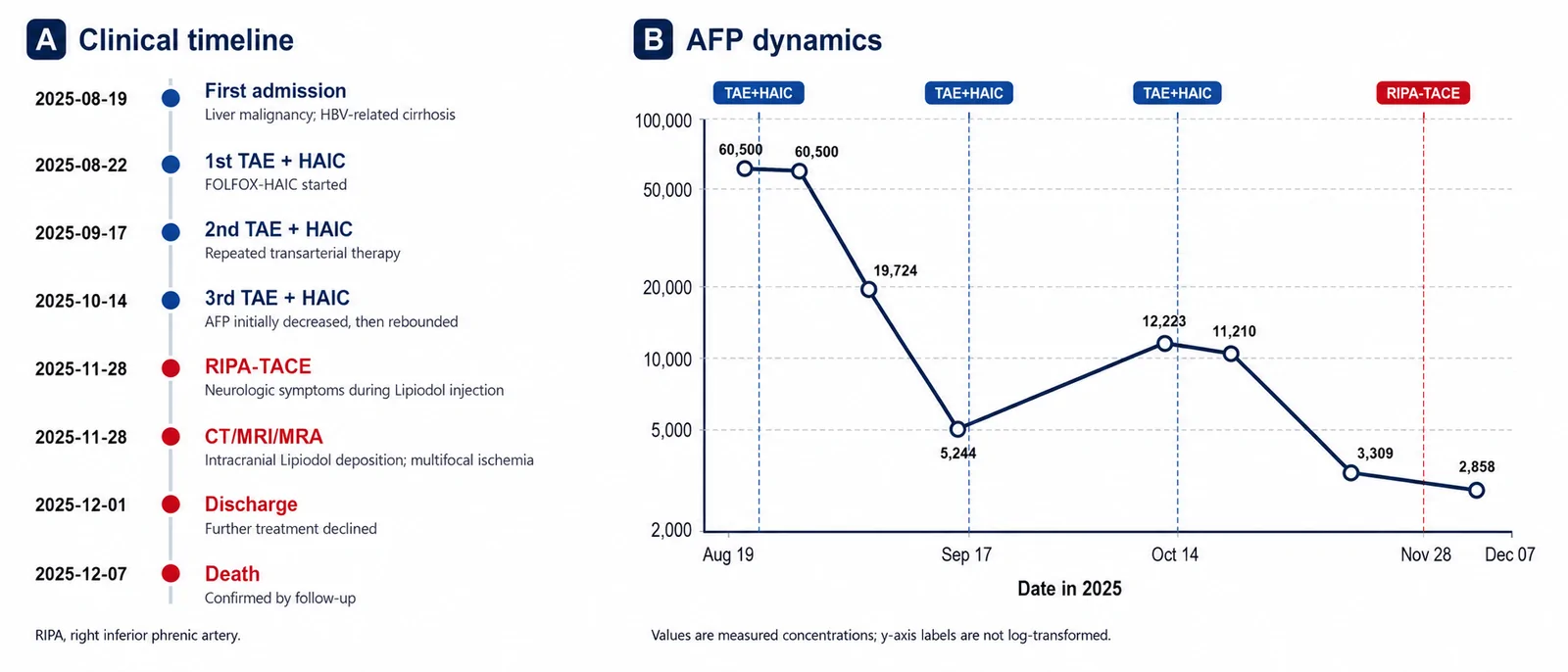
Treatment timeline and AFP dynamics. **(A)** Key clinical events, including initial admission, the first three transarterial treatments plus HAIC, the fourth right-inferior-phrenic-artery-related TACE, neuroimaging evaluation, discharge against medical advice, and death during follow-up. **(B)** AFP changes during treatment, shown on a logarithmic scale.

On August 22, 2025, the first interventional procedure was performed under local anesthesia. The middle hepatic artery and right hepatic arterial tumor-supplying branches were embolized with iodized oil and 50–150 um gelatin sponge embolic microspheres, followed by left hepatic arterial iodized-oil injection. A microcatheter was retained in the proper hepatic artery for continuous FOLFOX hepatic arterial infusion chemotherapy (HAIC). The second and third procedures were performed on September 17 and October 14, 2025, with repeat hepatic arteriography, iodized-oil embolization through hepatic arterial branches, and postoperative FOLFOX-HAIC. With tislelizumab and lenvatinib mesylate, AFP decreased from a peak of 60,500 ng/mL to 2,858 ng/mL (**Figure 1**).

**Figure 2:**
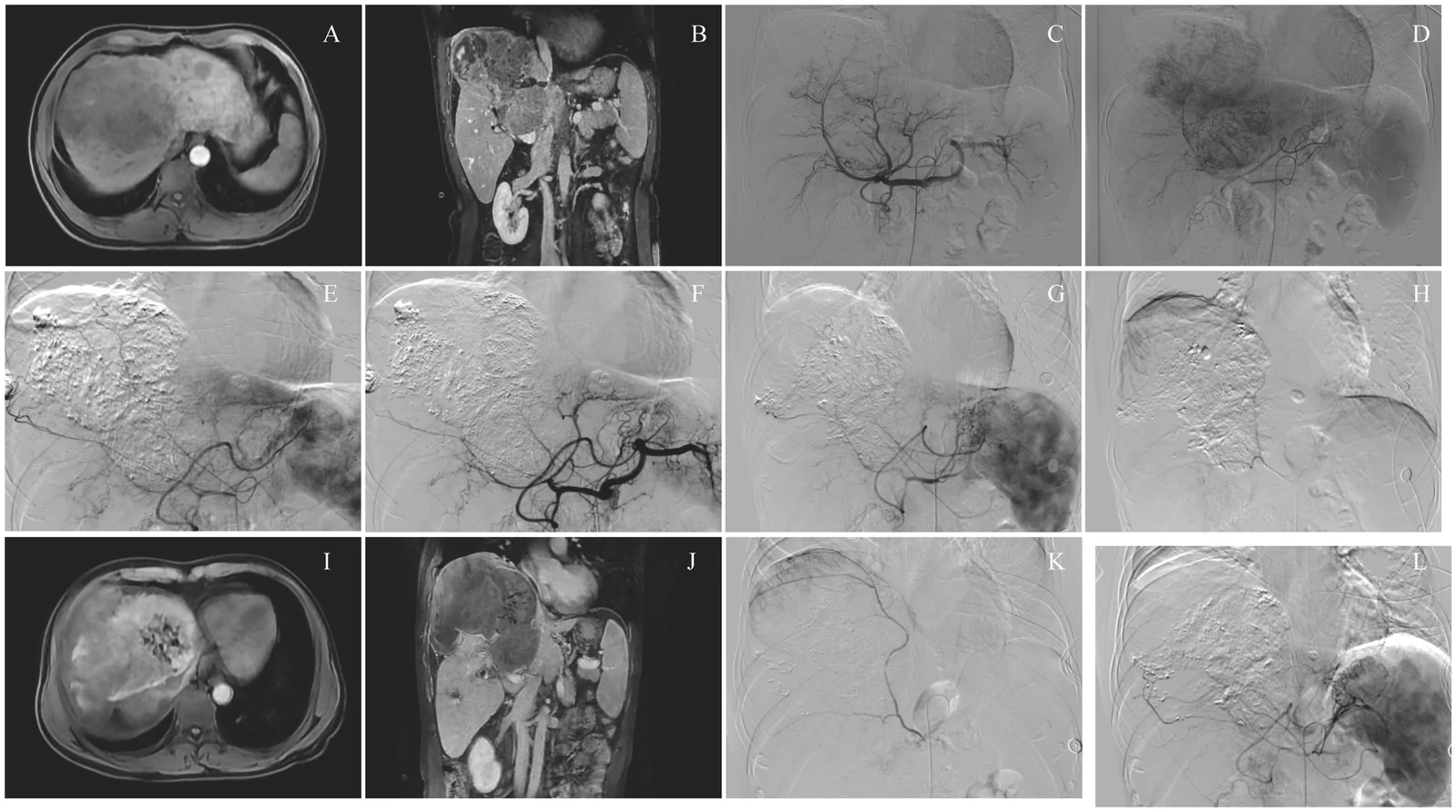
Hepatic images related to the four transarterial treatments. **(A–D)** First interventional procedure, showing the hepatic-dome tumor, tumor staining, and post-embolization changes. **(E, F)** Second procedure. **(G, H)** Third procedure. **(I–L)** Fourth procedure, showing residual or suspicious active lesion at the hepatic dome, right-inferior-phrenic-artery-related supply, and post-embolization changes.

The patient was readmitted on November 26, 2025. Routine preoperative transthoracic echocardiography showed no structural cardiac abnormality; agitated-saline contrast echocardiography (bubble study) and transesophageal echocardiography were not performed. On November 28, 2025, hepatic arteriography, right inferior phrenic arteriography, and TACE were performed. Celiac angiography showed post-treatment changes in the right hepatic lobe without definite new tumor staining or an arteriovenous fistula. Selective right inferior phrenic arteriography demonstrated patchy suspicious tumor staining at the hepatic dome, without a definite arteriovenous fistula or abnormal communication. Because the dominant hepatic arterial tumor supply had decreased after the first three treatments and the residual hepatic-dome tumor was supplied by the right inferior phrenic artery, this collateral route was selected for the fourth procedure. After superselective catheterization, an iodized-oil emulsion containing lobaplatin 10 mg, idarubicin 10 mg, and iodized oil 10 mL was administered. Quantitative injection pressure, injection rate, and fluoroscopy time were not documented in the available procedural record. Good intratumoral Lipiodol accumulation was observed (**Figure 2**).

Approximately eight minutes after the start of the procedure, while the iodized-oil emulsion was being injected, the awake patient suddenly developed flexion of both lower limbs and weakness of both upper limbs. He was initially conscious and answered appropriately, which made the neurological change directly observable rather than inferred from postoperative recovery. Limb weakness then rapidly progressed. The operator immediately stopped the injection and further embolization, removed the catheter and femoral sheath, and compressed the puncture site. Emergency cranial CT was obtained immediately, followed by MRI and MRA, creating a continuous procedural-to-imaging record of the event.

## Diagnostic assessment, intervention, follow-up, and outcomes

**Figure 3:**
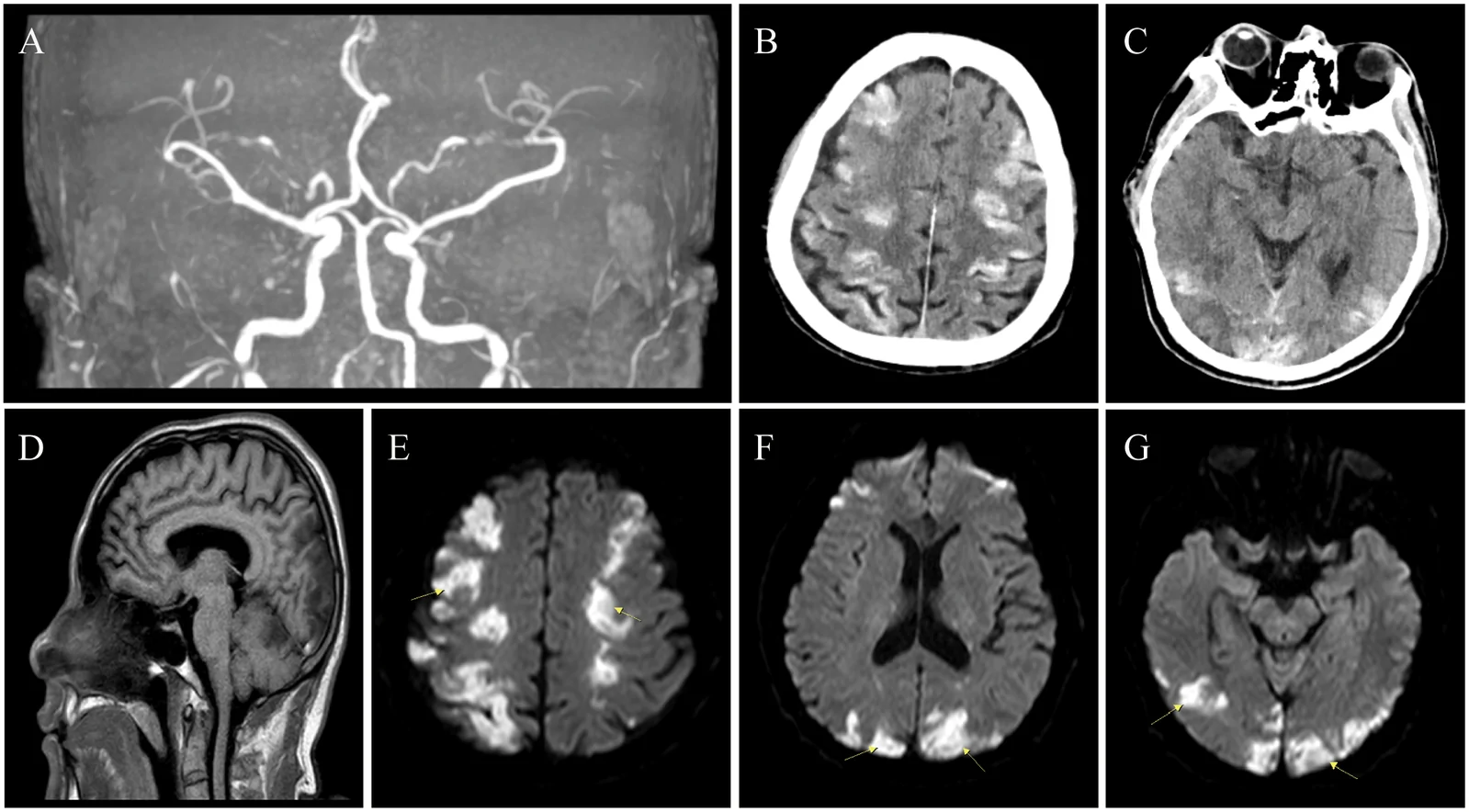
Brain imaging after intraoperative neurological deterioration. **(A)** MRA showing no definite major intracranial arterial occlusion. **(B, C)** Immediate cranial CT showing multiple hyperdense intracranial lesions; virtual non-contrast imaging supported iodized-oil deposition. **(D)** Sagittal MR image. **(E–G)** Diffusion-weighted MRI showing multifocal acute ischemic lesions in bilateral supratentorial and infratentorial regions.

Immediate cranial CT showed multiple hyperdense lesions in both cerebellar hemispheres, both thalami, periventricular regions, and the frontal, temporal, parietal, and occipital cortices (**Figure 3**). Most lesions disappeared on virtual non-contrast images, supporting intracranial iodized-oil deposition rather than hemorrhage (16). Diffusion-weighted MRI showed multifocal acute ischemic lesions involving the bilateral cerebellar hemispheres, thalami, and cerebral cortices. MRA demonstrated sparse distal branches but no definite occlusion of the major intracranial arteries; this excluded a treatable large-vessel occlusion but did not exclude distal arteriolar or penetrating-vessel obstruction. Based on the synchronized intra-procedural onset, immediate CT and virtual non-contrast findings, multifocal diffusion restriction, and absence of major arterial occlusion, TACE-related CLE was diagnosed.

**Figure 4:**
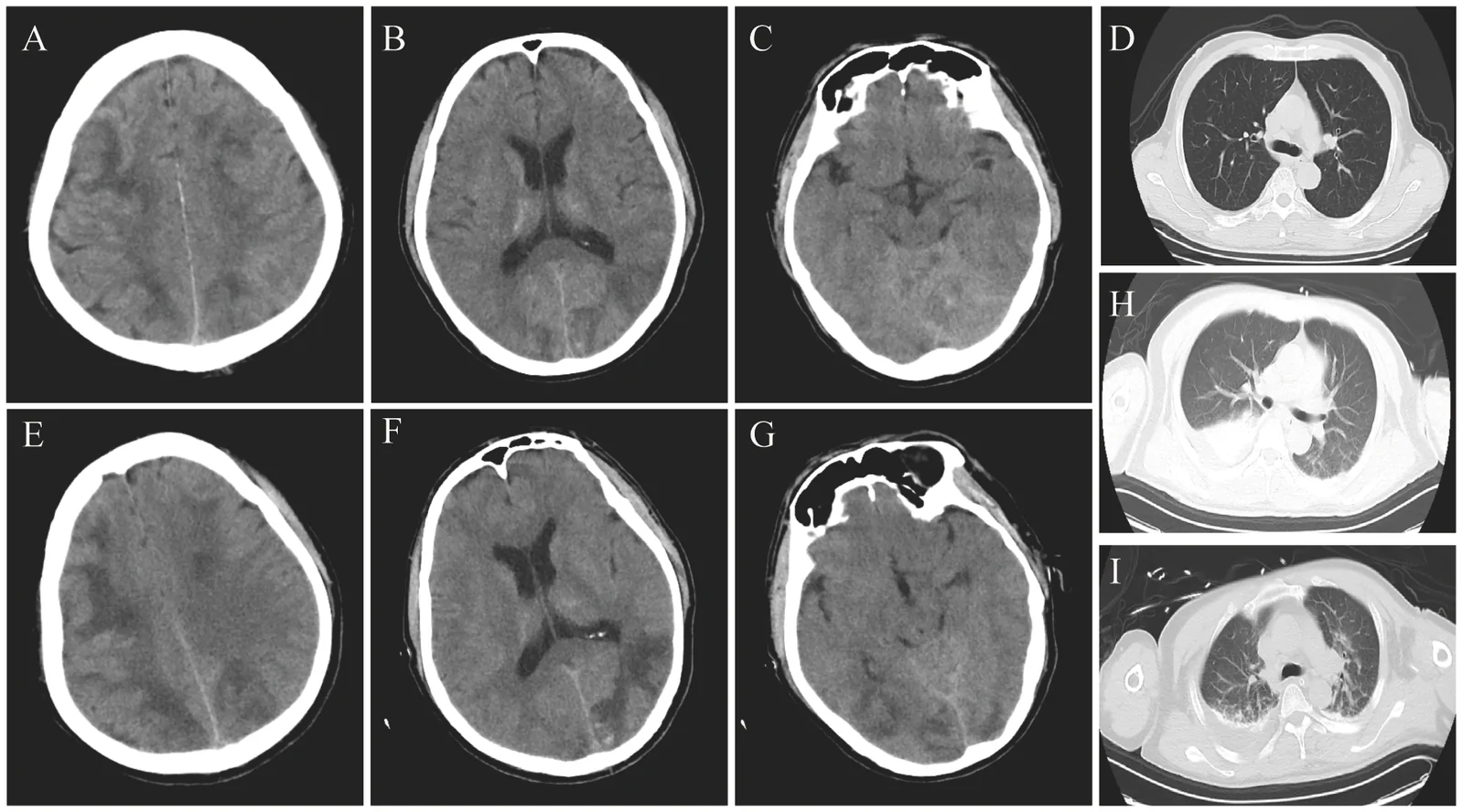
Postoperative follow-up imaging. **(A–C)** Cranial CT on postoperative day 1 showing decreased intracranial hyperdense Lipiodol deposits. **(E–G)** Cranial CT on postoperative day 3 showing further clearance of deposits while ischemic changes persisted. **(D)** Preprocedural comparison image before the fourth interventional treatment. **(H, I)** Chest CT on postoperative days 1 and 3 showing right lower-lobe atelectasis, pleural effusion, and iodized-oil deposition in the treated hepatic region.

After the event, the patient received continuous monitoring, head-up positioning, low-molecular-weight heparin calcium, high-dose corticosteroids, medium- and long-chain lipid emulsion, butylphthalide, edaravone, fluid therapy, and supportive treatment. Neurology and external interventional oncology consultations were obtained. Mechanical thrombectomy or endovascular embolus retrieval was considered but not pursued because MRA showed no targetable large-vessel occlusion and the imaging pattern was consistent with diffuse microembolization. Consciousness deteriorated from alertness to somnolence, limb strength disappeared, and the patient was transferred to the neurological care unit. CT on postoperative day 1 showed a reduction in intracranial high-density iodized-oil deposits, and CT on postoperative day 3 showed further clearance (**Figure 4**). However, this radiologic improvement was discordant with the clinical course: multifocal ischemic injury persisted, consciousness and motor function did not recover, and the patient remained severely neurologically impaired. Chest CT showed iodized-oil deposition in the treated hepatic region, right lower-lobe atelectasis, and small pleural effusion. Follow-up with the family confirmed that the patient died at home on December 7, 2025. No autopsy was performed, and no formal brain-death determination was made. Because death occurred at home after discharge against medical advice, the exact terminal cause and mode of death could not be independently ascertained. Based on the documented clinical course and neuroimaging findings, CLE-related multifocal cerebral infarction and severe ischemic brain injury were considered the most likely contributors to death.

## Discussion

The incremental value of this report lies in its evidence chain and clinical phenotype: repeated liver-directed therapy with an apparent tumor-marker response; residual hepatic-dome disease supplied by the right inferior phrenic artery; no visible shunt or vascular lake on angiography; neurological collapse during active injection; CT and virtual non-contrast evidence of intracranial iodized-oil deposition; diffusion-weighted MRI-confirmed multifocal acute ischemia; no major intracranial arterial occlusion; and a fatal outcome despite serial radiologic Lipiodol clearance. This pattern differs from reports characterized by visible vascular lakes or shunts, delayed neurological presentation, or subsequent recovery (9, 13). A recent review identified death in 15 of 49 reported CLE cases (13). Compared with those fatal cases, the present case is notable for intra-procedural symptom onset, immediate multimodal imaging confirmation, and serial Lipiodol clearance without neurological improvement.

The temporal sequence is important for interpreting the event. The patient was awake under local anesthesia, neurological symptoms were observed during emulsion delivery, the injection was stopped immediately, and brain imaging followed without delay. Hyperdense lesions that disappeared on virtual non-contrast images supported iodine-compatible material rather than hemorrhage. Diffusion-weighted MRI then showed widespread acute ischemic injury in multiple vascular territories, whereas MRA showed no major intracranial arterial occlusion. Together, these findings are most compatible with intracranial Lipiodol microembolization followed by multifocal ischemic injury, although the exact embolic route cannot be demonstrated directly. Similar symptoms and imaging patterns have been reported in previous CLE cases (7–13).

Follow-up imaging highlights an important distinction between transient Lipiodol deposition and permanent ischemic injury. Intracranial iodized-oil deposits decreased markedly on serial CT, but DWI-confirmed ischemic lesions and severe neurological deficits persisted. This discordance suggests that neurological outcome was driven mainly by the downstream burden of microvascular ischemic injury rather than by the persistence of visible Lipiodol deposits. Therefore, radiologic clearance of Lipiodol should not be interpreted as evidence of neurological recovery.

In this patient, the fourth procedure targeted the right inferior phrenic artery because residual hepatic-dome tumor staining was supplied by this collateral route after three previous liver-directed treatments. The right inferior phrenic artery is a common extrahepatic collateral supply for HCC at the hepatic dome, and extrahepatic collateralization becomes more likely after repeated transarterial therapy (17–19). Although treatment through this artery is generally feasible, it may be associated with chest pain, pleural effusion, atelectasis, pulmonary Lipiodol deposition, and systemic-to-pulmonary shunting (17, 19). Therefore, negative routine angiography may not be sufficient to exclude occult embolic pathways in heavily pretreated hepatic-dome tumors.

Several mechanisms may have contributed, but none can be proven from the available data. Tumor-related microarteriovenous shunts may be too small, intermittent, or slow-flow to be detected by angiography, but may open during emulsion injection. A vascular lake with venous drainage is another possibility (9). Intracardiac or intrapulmonary right-to-left shunting may also permit systemic embolization. Routine transthoracic echocardiography showed no structural cardiac abnormality, but no agitated-saline contrast study or transesophageal echocardiography was performed. Consequently, a patent foramen ovale or another occult right-to-left shunt was not excluded and remains a possible route of paradoxical embolization. Conventionally prepared Lipiodol emulsions generally contain droplets of approximately 30-160 µm, depending on preparation (20). Previous CLE reports have proposed that a much smaller lipid-globule fraction (<7 µm) may traverse the pulmonary microcirculation (8, 9), a dimension comparable to the reported mean human pulmonary capillary diameter of approximately 6.3 µm (21). Because droplet size was not measured in this case, direct transpulmonary passage remains hypothetical. Pulmonary Lipiodol embolism after TACE has been described (22), but this patient’s chest CT report described iodized-oil deposition only in the treated hepatic region; the associated atelectasis and pleural effusion did not provide direct evidence of pulmonary Lipiodol embolization.

A right-inferior-phrenic-artery-related pathway is plausible but remains hypothetical. This artery supplies the diaphragm and may participate in systemic-to-pulmonary communications. In a prospective C-arm CT study, a systemic-to-pulmonary shunt from the right inferior phrenic artery was observed in 22 of 32 patients (69%) (19), and right inferior phrenic artery involvement has also been described in CLE cases (13). Repeated TACE, inflammation, adhesions, or tumor invasion near the diaphragm could promote small communications that remain occult on routine DSA. Under injection pressure, such channels might permit embolic material to reach the pulmonary circulation and, if an additional right-to-left pathway were present, the systemic circulation. However, no such pathway was demonstrated angiographically in this patient; this explanation should therefore be regarded as a hypothesis rather than a proven mechanism.

The major lesson is preventive and relates to case selection as well as injection technique. In patients with repeated transarterial treatment, hepatic-dome tumors, and right inferior phrenic artery collateralization, iodized-oil emulsion should be treated cautiously even when angiography is negative. Practical safeguards include reviewing prior angiograms for evolving collateralization, considering C-arm CT or additional angiographic runs when feasible, injecting slowly in small divided aliquots, pausing after early Lipiodol deposition, and maintaining direct communication with the awake patient during local anesthesia. Sudden cough, hypoxemia, limb movement, weakness, or altered consciousness during injection should be regarded as an embolic warning sign until proven otherwise. Injection should stop immediately, and early CT with virtual non-contrast or dual-energy techniques, followed by diffusion-weighted MRI, ADC mapping, and MRA, can help distinguish iodized-oil deposition, acute infarction, and hemorrhage (16). This single case is limited by the lack of complete dynamic DSA video, C-arm CT, contrast-enhanced chest CT, agitated-saline contrast echocardiography, transesophageal echocardiography, formal cardiopulmonary shunt testing, measured Lipiodol droplet size, autopsy confirmation, and independent ascertainment of the terminal cause and mode of death. The exact embolic route and terminal mechanism therefore remain inferential.

## Conclusion

This case describes fatal CLE during right inferior phrenic artery TACE despite negative angiographic findings. The case emphasizes that negative angiography does not exclude occult embolic pathways. After CLE occurs, radiologic clearance of intracranial Lipiodol should not be equated with neurological recovery, because DWI-confirmed ischemic injury may determine outcome.

## Data Availability

The original contributions presented in the study are included in the article. Further inquiries can be directed to the corresponding author. Additional patient-level data are not publicly available because of privacy and ethical restrictions.
